# Large anisotropy of ferroelectric and pyroelectric properties in heteroepitaxial oxide layers

**DOI:** 10.1038/s41598-018-22349-y

**Published:** 2018-03-12

**Authors:** R. Moalla, S. Cueff, J. Penuelas, B. Vilquin, G. Saint-Girons, N. Baboux, R. Bachelet

**Affiliations:** 1 Institut des Nanotechnologies de Lyon (INL) - CNRS UMR 5270, Univ. Lyon, Ecole Centrale de Lyon, Bâtiment F7, 36 av. Guy de Collongue, 69134 Ecully Cedex, France; 2 Institut des Nanotechnologies de Lyon (INL) - CNRS UMR 5270, Univ. Lyon, INSA de Lyon, Bâtiment Blaise Pascal, 7 avenue Jean Capelle, 69621 Villeurbanne Cedex, France

## Abstract

Epitaxial PbZr_0.52_Ti_0.48_O_3_ (PZT) layers were integrated on Si(001) with single PZT {001} orientation, mosaïcity below 1° and a majority of *a*-oriented ferroelectric domains (∼65%). Ferroelectric and pyroelectric properties are determined along both the out-of-plane and in-plane directions through parallel-plate capacitor and coplanar interdigital capacitor along the <100>_PZT_ direction. A large anisotropy in these properties is observed. The in-plane remnant polarization (21.5 µC.cm^−2^) is almost twice larger than that measured along the out-of-plane direction (13.5 µC.cm^−2^), in agreement with the domain orientation. Oppositely, the in-plane pyroelectric coefficient (−285 µC.m^−2^.K^−1^) is much lower than that measured out-of-plane (−480 µC.m^−2^.K^−1^). The pyroelectric anisotropy is explicated in term of degree of structural freedom with temperature. In particular, the low in-plane pyroelectric coefficient is explained by a two-dimensional clamping of the layers on the substrate which induces tensile stress (from thermal expansion), competing with the decreasing tetragonality of *a*-domains (shortening of the polar *c*-axis lattice parameter). Temperature-dependent XRD measurements have revealed an increased fraction of *a*-domains with temper*a*ture, attesting the occurrence of a partial two-dimensional clamping. These observed properties are of critical importance for integrated pyroelectric devices.

## Introduction

Some of the most prominent features offered by perovskite oxides are ferroelectricity, piezoelectricity, pyroelectricity and ferromagnetism, which are exploited in a wide range of applications^1–3^. Particularly, all ferroelectric oxides simultaneously present pyroelectric and piezoelectric properties that can be exploited in various devices, such as non-volatile memories, sensors, actuators and energy harvesters^4–6^. These functional properties strongly depend on the oxide’s structure, especially on its tetragonality, and on its ferroelectric domain structure which determines the distribution of polarization axis orientations. In most applications, these functional oxides must be integrated as thin films. However, their resulting properties can strongly differ from that of bulk material given that thin film growth alters the material texture. In particular, epitaxial thin film growth could be leveraged to control and enhance ferroelectric properties through the fabrication of single crystal materials with controlled ferroelectric domain structure^7^.

Yet, these benefits have not been clearly demonstrated experimentally, as it requires a careful and complex assessment of the impact of epitaxy on potentially anisotropic physical properties. Attempts to investigate such a “functional” anisotropy have been proposed, for instance by varying the substrate orientation^8–10^, or by tailoring the epitaxial strain using different substrates^11–13^, but so far, no clear anisotropy measurements (probed along different crystallographic axes on the same oriented film) have been reported at the macroscale.

Studies of ferroelectric oxide thin films are usually focused on out-of-plane characterizations using parallel plate capacitors; in-plane investigations being so far very limited. Some dielectric and ferroelectric in-plane studies were conducted using interdigital capacitors (IDCs) for a variety of technological applications such as microwave integrated circuits^14^, surface acoustic wave (SAW) devices^15^ and chemical sensors^16^. In-plane dielectric properties have thus been reported for polycrystalline barium strontium titanates (BST)^17,18^ and epitaxial PMN-PT films^19^, and in-plane ferroelectric properties have been reported for epitaxial BST films on different oxide substrates^20–22^. However, these reports do not include out-of-plane characterizations and do not contain enough experimental details for parameters extraction to evidence a possible anisotropy. Furthermore, they do not include measurements of the in-plane pyroelectric response.

Although less studied than dielectric, ferroelectric and piezoelectric properties, pyroelectric properties, coupling a polarization variation with temperature, can lead to a broad range of applications, most notably thermal/IR sensing, imaging and thermal energy harvesting^5,23^. Pb(Zr,Ti)O_3_ (PZT) is a prototypical ferroelectric oxide that exhibits the largest reported polarizations, piezoelectric and pyroelectric coefficients^4,5^. Indeed, it has been used for instance to fabricate ferroelectric random access memories (FRAM)^4,24^, piezoelectric actuators^25^, mechanical energy harvesters^6^, pyroelectric nanogenerators for driving wireless sensor networks (WSNs)^26^, and enhanced nanogenerators based on coupled properties^27^. Moreover, thanks to its perovskite structure, PZT can be monolithically integrated on Si by epitaxy, *via* a SrTiO_3_ (STO) buffer layer^13,28,29^. Noticeably, a recent report has shown that epitaxial PZT layers grown on STO/Si templates lead to a gain of two orders of magnitude in pyroelectric energy conversion with respect to their polycrystalline counterparts^30^.

In this paper, we report both out-of-plane and in-plane ferroelectric and pyroelectric measurements of PZT films, epitaxially integrated on silicon, and demonstrate a large anisotropy in these functional properties, correlated with their structural properties.

## Results and Discussion

**Figure 1:**
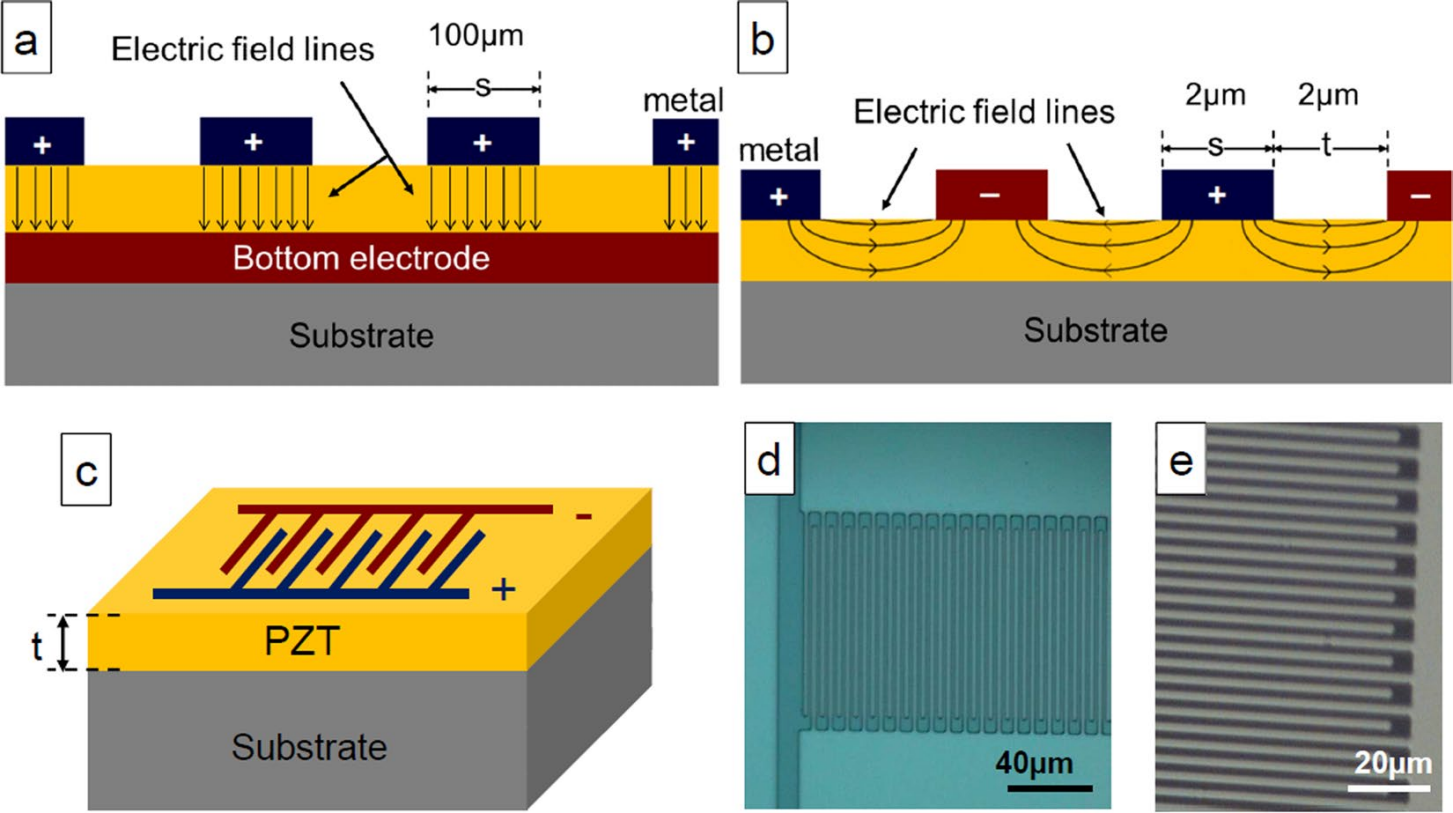
(**a**–**c**) Sketchs of the orientation of field lines in (**a**) parallel-plate capacitors, and (**b**,**c**) coplanar interdigital capacitors (IDCs). (**d**–**e**) Optical images of a Pt-patterned IDCs formed by photolithography.

Five hundred nanometers thick epitaxial PZT (52:48) layers were grown by sol-gel process on silicon (001) substrate buffered with ∼10 nm thick epitaxial STO layers grown by oxide molecular beam epitaxy (MBE)^31^. Two different architectures were investigated: *i)* PZT layers grown on top of a 30 nm thick SrRuO_3_ (SRO) bottom electrode layer grown by radio frequency (rf) magnetron sputtering on top of STO/Si for out-of-plane (OOP) characterizations [Fig. 1(a)], and *ii)* PZT layers directly grown on top of the STO/Si pseudo-substrate for in-plane (IP) characterizations [Fig. 1(b,c)]. More details on the growth process can be found in the Method section and in previous reports^13,30^. Two configurations of platinum top electrodes were elaborated by rf magnetron sputtering at room temperature, UV-lithography and lift-off process for further electrical OOP and IP characterizations, and are sketched in Fig. 1 (For the out-of-plane (OOP) measurement in a metal-ferroelectric-metal (MFM) structure, the electric field lines pass across the PZT layer vertically toward the bottom electrode, the properties thus obtained, such as P_r_, E_c_, *p* etc, are those of the layer in the direction perpendicular to the surface. For in-plane (IP) measurement, two successive fingers belong to two different combs inversely polarized. Due to the absence of the lower conductive layer, the field lines pass through the PZT layer horizontally from one finger to the next. Properties thus extracted are those of the layer in the direction parallel to the surface. We consider that one of the two combs is the equivalent of the upper electrode of a plate capacitor and the second is the equivalent of the lower electrode, so the distance between two successive fingers is equivalent to the thickness of the layer between the electrodes. The total area of the capacitor is considered as the number of fingers in a comb multiplied by the surface of a finger). Square-shaped Pt top-contacts form the parallel plate electrodes on top of the PZT/SRO/STO/Si (001) heterostructure for OOP measurements [Fig. 1(a)]. Interdigital Pt top electrodes form the coplanar IDCs on the top of PZT/STO/Si (001) heterostructure along the <100>_PZT_ direction for the IP measurements [Fig. 1(b,c)]. The pattern includes 50 fingers, with two successive fingers belonging to two different combs inversely polarized. The finger length and width is of 100 µm and 2 µm, respectively, and inter-finger gap is 2 µm [Fig. 1(d,e)].

**Figure 2:**
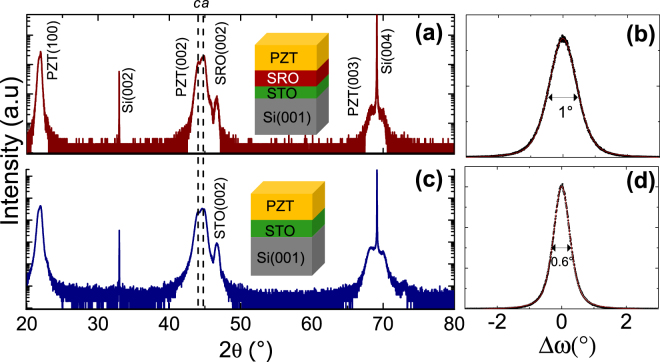
(**a**,**c**) XRD θ/2θ scans of PZT deposited on (**a**) SRO/STO/Si(001) and (**b**) STO/Si(001). Dash lines show the {002} bulk Bragg reflections of *a*-oriented and *c*-oriented PZT ferroelectric domains. (**b**,**d**) XRD ω-scans around the {002} Bragg reflections of PZT on (**b**) SRO/STO/Si(001) and (**d**) STO/Si(001).

The XRD θ/2θ scans recorded before the deposition of the top electrodes on both samples shown in Fig. 2(a,c) attest to similar crystalline orientations and structures of the PZT layers. Bragg diffraction peaks corresponding to {00 l} reflection only of PZT, SRO, STO and Si are observed, indicating fully {00 l}-oriented heterostructures. The same peak positions are observed on both samples, indicating the same structure and global strain state. Pseudo-tetragonal *a*- and *c*-oriented domains of the PZT layer can be discriminated by fitting the {002} Bragg peak of PZT using two contributions. The PZT layer is mainly *a*-oriented (∼65%) here due to thermal expansion mismatch with respect to the Si substrate, as explained elsewhere^13^. The corresponding OOP and IP cell parameters are 4.05 Å and 4.12 Å for the *a*-oriented domains, and 4.11 Å and 4.10 Å for the *c*-oriented domains, respectively^13^. The structural details can be found elsewhere^13^. The domain size is expected to be in the range of hundred nanometers from the Landau-Lifshitz-Kittel scaling^32^. The epitaxial relationship between the oxide layers and the silicon substrate was previously measured as [100]PZT(001)//[100]SRO(001)//[100]STO(001)//[110]Si(001)^13,33^. The out-of-plane mosaicity of the PZT layers measured on the (002) Bragg reflection is below 1°, and slightly better for the PZT/STO/Si heterostructure (∼0.6°) compared to the PZT/SRO/STO/Si heterostructure [Fig. 2(b,d)]. The small difference could be due to the difference between the STO/Si templates quality or to the presence of additional SRO layer in the second heterostructure.

**Figure 3:**
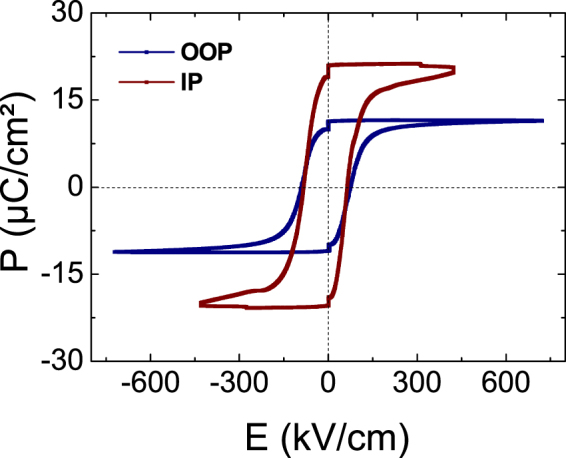
Ferroelectric hysteresis loops (Polarization versus electric field by PUND method) of PZT epitaxial layers measured along the out-of-plane (OOP) and in-plane (IP) <100> direction at room temperature.

The ferroelectric hysteresis loops (P-E) measured macroscopically along both the OOP and IP directions with the positive-up negative-down (PUND) pulse train method are presented in Fig. 3. In the configuration of coplanar electrodes, the electric field can penetrate into the substrate and thus could affect the results. Here, the low dielectric constant of the Si substrate (11.68), compared to those of high-*k* oxides such as PZT, reduces the electric field penetration in the substrate. It is worth noting that the coercive field characterizing the switching of ferroelectric domains is the same measured in the IP and OOP configurations, which gives us confidence in our measurements. The remnant polarization (P_r_) measured along the IP direction (21.5 μC.cm^−2^) is much larger than that measured along the OOP direction (13.5 μC.cm^−2^), by a factor of almost 2. These results are in agreement with the ferroelectric domain structure of the PZT layers, dominated here by *a*-oriented domains on Si(001)^13^. The IP over OOP polarization ratio roughly matches *a*-oriented over *c*-oriented ferroelectric domains concentration estimated from X-ray diffracted intensities.

**Figure 4:**
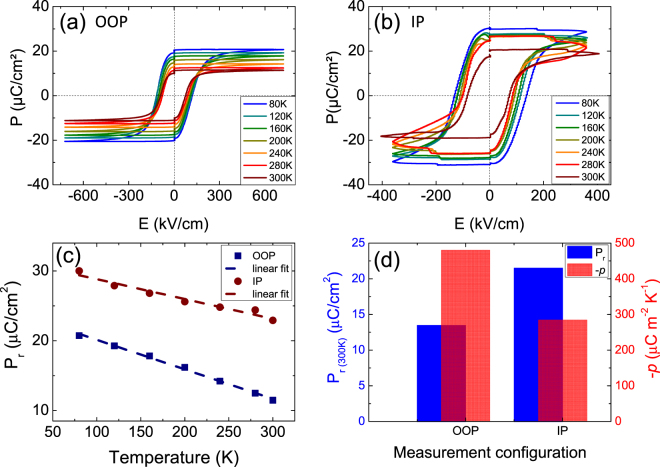
(**a**–**b**) Ferroelectric hysteresis loops (Polarization versus electric field by PUND method) of the PZT epitaxial layers measured at various stabilized temperatures from 80 K to 300 K (**a**) along the out-of-plane (OOP) direction, and (**b**) along the in-plane (IP) 〈100〉 direction. (**c**) Variation of the corresponding remnant polarization as a function of temperature. (d) Summary of the remnant polarization and the pyroelectric coefficient measured both along the OOP and IP directions.

Pyroelectric coefficients (*p*), which link a change of the remnant polarization with a temperature variation^5,23^, were extracted from PUND ferroelectric hysteresis loops recorded in both configurations at different stabilized temperatures ranging here from 80 K to 300 K [Fig. 4(a,b)]. As expected, the remnant polarization decreases when the temperature increases in both cases^5^. The variation of P_r_ as a function of temperature, shown in Fig. 4(c) for both configurations, is linear in this temperature range. It is worth noting that the same linear slope is observed at least up to 400 K along the OOP direction^13^, that means a constant pyroelectric coefficient in this whole temperature range (80 K–400 K), which is consistent with the fact that we are working far from the Curie temperature T_c_ (polarization vs. temperature plot exhibits a quasi-linear behavior). A possible monoclinic to tetragonal phase transition occurring around room temperature in bulk material at this PZT composition^34^, is shifted at higher temperatures than 500 K in epitaxial films on Si^33^, and would be barely measurable electrically because of slight structural difference of less than 0.5° in the *c*-axis orientation with respect to the normal of the *a-b* plane^34^. The decrease of P_r_ with temperature is much lower along the IP direction than along the OOP direction, despite a large IP polarization and tetragonality. The resulted IP and OOP pyroelectric coefficients extracted from these indirect measurements (*p* = ΔP_r_/ΔT) are −285 and −480 μC m^−2^ K^−1^, respectively. These measurements exclude the extrinsic pyroelectric effect due to domain wall motion with temperature^12,30^. Although these coefficients are of the same order of magnitude than those reported in previous studies,^13,30^ they differ from each other by a factor of almost 2. They differ from a factor of almost 3 if they are normalized with respect to their remnant polarization at room temperature (*p*/P_r_). The remnant polarizations (P_r_) and the pyroelectric coefficients (*p*) measured along both directions are compared in Fig. 4(d).

**Figure 5:**
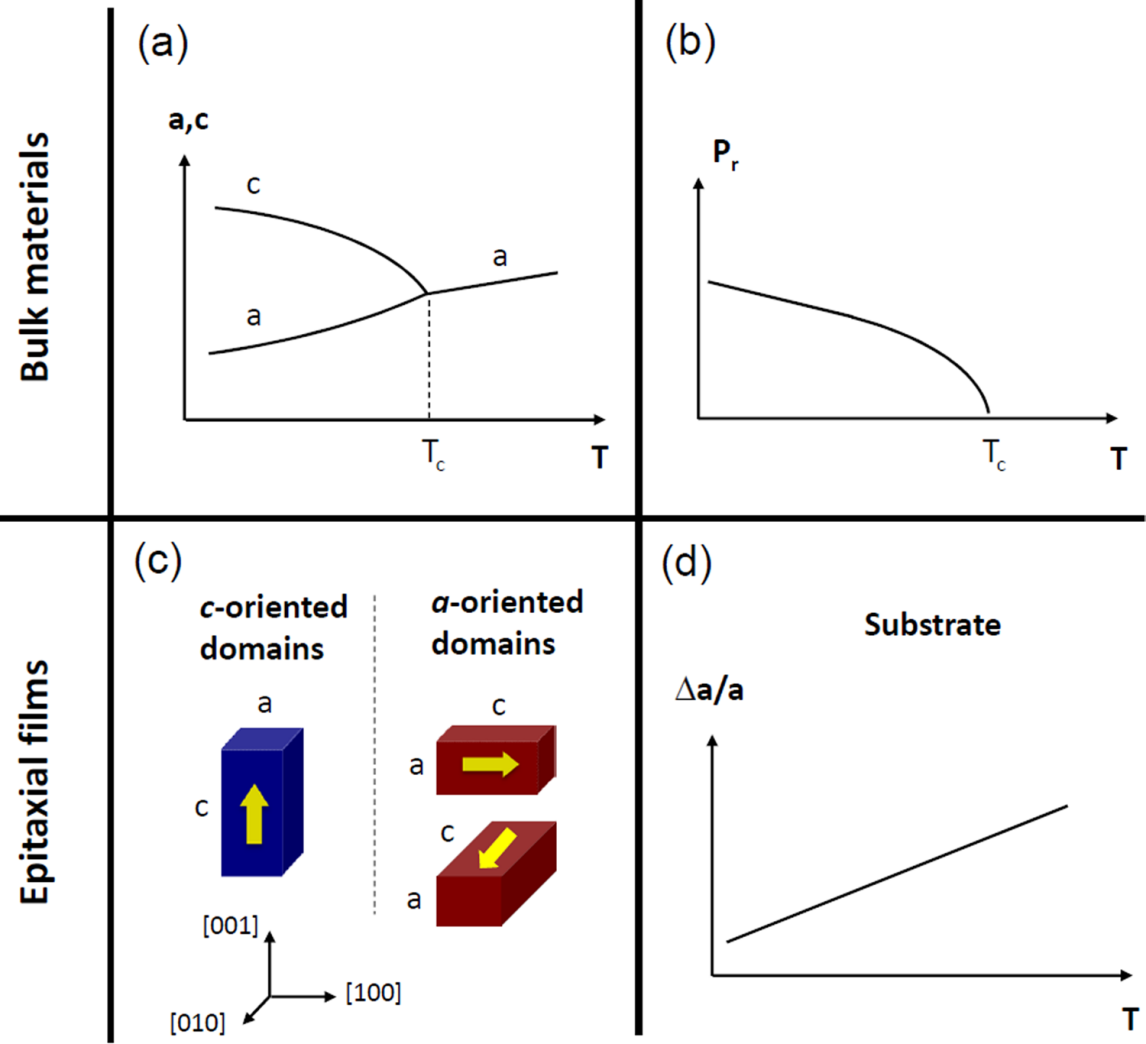
Some sketched basic features of ferroelectric materials, in bulk form (**a**,**b**) and epitaxial films (**c**,**d**), to take into account for ferroelectric and pyroelectric anisotropy. (**a**) Variation of the lattice parameters with temperature. (**b**) Consequent variation of remnant electric polarization with temperature, leading to pyroelectricity. (**c**) The main orientations of the tetragonal domains, leading to different polydomain structures. (**d**) Thermal expansion of the substrate.

The *primary* pyroelectric effect (polarization variation with temperature) is coupled with the *secondary* pyroelectric effect (crystal deformation *via* piezoelectric effect)^23^. The secondary pyroelectric effect is important in PZT since its piezoelectric coefficient is high^6^. The reduction of tetragonality with temperature by shortening the lattice parameter of the polar *c*-axis [Fig. 5(a)] leads to the decrease of remnant polarization [Fig. 5(b)]^29^, which is responsible for the pyroelectricity^5,23^. The variation of the polarization with temperature should be larger along the main polarization and the largest tetragonality [Fig. 5(c)]^12,29^. Even if both the main polarization (*c*-axis) and the largest tetragonality lie in-plane here, the OOP pyroelectric response is much larger than the IP pyroelectric response. This can be explained structurally by a two-dimensional clamping of the epitaxial layers to the substrate which tends to impose its thermal expansion (tensile stress) [Fig. 5(d)]^13,29,33^, competing with the reduction of tetragonality (decrease of *c*-axis lattice parameter of *a*-domains)^29,34^. The thermal expansion coefficient of the Si substrate is around 3 × 10^−6^ K^−1^^13,29^, whereas the variation of the *c*-axis parameter with temperature (Δc/c) in the tetragonal phase far from T_c_ is negative in the range of −4 × 10^−5^ K^−1^ in bulk PZT^34^. The IP structural conflict with temperature here is so great that the clamping seems to be only partial. In case of pure clamping, the IP pyroelectric coefficient would be of positive sign (increase of tetragonality and consequent increase of P_r_ along the IP direction). And, in case of absence of clamping, the IP pyroelectric coefficient would be larger (in absolute value) than that OOP.

**Figure 6:**
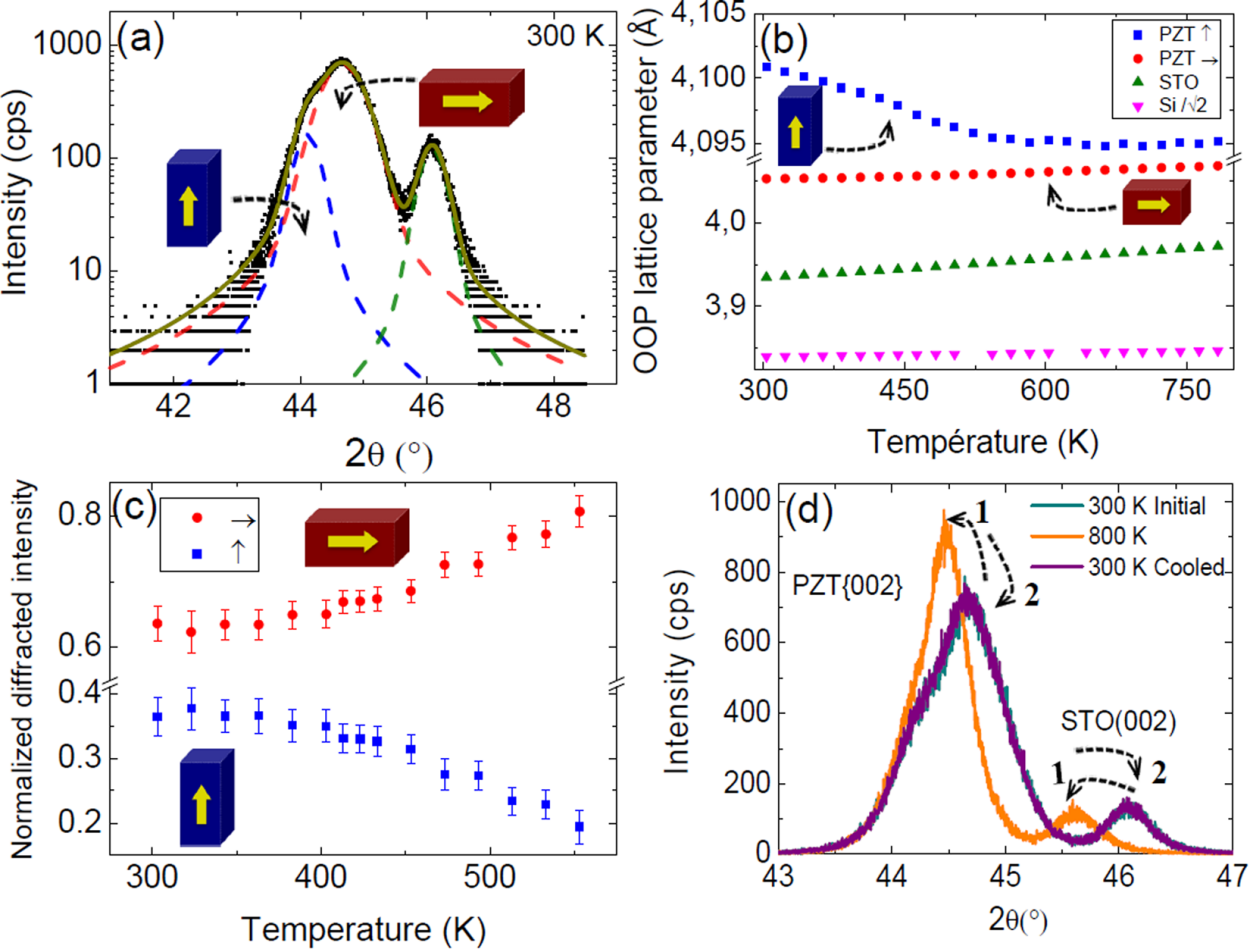
(**a**) XRD θ/2θ scan of {002} Bragg peak of PZT at room temperature and fits corresponding to *a*-oriented and *c*-oriented domains components. (**b**) Out-of-plane plane lattice cell parameters as a function of temperature. (**c**) Normalized diffracted intensity of *a*-oriented and *c*-oriented ferroelectric domains as a function of temperature. (**d**) XRD θ/2θ scan of {002} Bragg peak of PZT at room temperature before the measurement at temperature, at 800 K, and at room temperature after cooling.

In order to assess the hypothesis of partial clamping effect, temperature-dependent XRD measurements were carried out on both samples. Both *a*- and *c*-oriented domains are measured [Fig. 6(a)]. The evolution of the cell parameters with temperature are in agreement with previous reports on epitaxial PZT layers on STO and Si (001) substrates [Fig. 6(b)]^29,33,35^. More interestingly, the evolution of the normalized diffracted intensity of these two peaks with temperature well below T_c_ shows that the *a*-oriented domains fraction increases at the expense of *c*-oriented domains with the temperature [Fig. 6(c)]. This observation evidences the existence of the partial clamping effect occurring to the PZT layers. The thermal expansion of the Si substrate forces the *c*-domains, having low tetragonality (close OOP and IP parameters), to become *a*-domains by IP tension with temperature, in agreement with theoretical results^36^. The variation of the domain fraction is not a prerequisite to obtain a lower IP pyroelectric coefficient than that OOP, but is a proof of the occurrence of a clamping effect, explaining the pyroelectric anisotropy here. The variation of the domain fraction seems to be monotone close to room temperature and enhanced with temperature within the error bars. That would mean that the two-dimensional clamping effect is enhanced with temperature, probably because of better matching of the PZT lattice with the thermal expansion of the Si substrate at higher temperature. More generally, this can be due to different temperature-dependant variations of the cell parameters and to complex equilibrium in the polydomain PZT epitaxial layer considering strain, domain structure and elastic energy^36–38^. This observation signals that the IP pyroelectric coefficient can be smaller (in absolute value) above 400 K or even of positive sign, enhancing even more the pyroelectric anisotropy. It is worth noting that this phenomenon is reversible (or purely elastic), meaning that the PZT layer and structure is not affected by the measurement at relatively high temperature [Fig. 6(d)].

To summarize, along the IP direction, two opposite forces occur when increasing the temperature, namely *i)* a shortening of the polar *c*-axis lattice parameter of *a*-domains which decreases the polarization by lowering the tetragonality, and *ii)* an extension induced by the thermal expansion of the substrate which can increase the *a*-domains over *c*-domains fraction^36^. These two main counterbalanced phenomena tend to minimize the variation of the polarization and thus the global IP pyroelectric coefficient. The degree of structural freedom along the OOP direction is larger than that IP, yielding a larger pyroelectric coefficient, inducing the pyroelectric anisotropy observed here. This shows that epitaxial effects strongly affect the pyroelectric response of the thin layers. More generally, the present study highlights the complex links between epitaxy and the resulting functional properties of oxide layers, and may be generalized to many cases where the functional properties depend on the ferroelectric domain structure, as the case of electro-optical properties for instance^39^. These results are essential to further tailor the pyroelectric properties of integrated films and to optimize the design of ferroelectric and pyroelectric nanodevices.

## Conclusion

To conclude, the large anisotropy of the ferroelectric and pyroelectric properties of epitaxial PZT layers integrated on silicon was demonstrated. In-plane remnant polarization is about 21.5 μC.cm^−2^ compared to 13.5 μC.cm^−2^ measured along the out-of-plane direction, in very good agreement with structural properties (65% of *a*-oriented domains). In-plane pyroelectric coefficient along the <100> direction is found lower than that measured out-of-plane (−285 µC.m^−2^.K^−1^ and −480 µC.m^−2^.K^−1^, respectively) oppositely to the main polar axis orientation. The pyroelectric anisotropy has been explained by the degree of structural freedom: two opposite in-plane forces occur with temperature (reduction of tetragonality of *a*-domains and thermal expansion imposed by the substrate) which minimizes the pyroelectric effect along the in-plane direction. These properties can be exploited in other ferroelectric/pyroelectric heterostructures for which the functional properties depend on their domain structure.

## Methods

### Elaboration

Single-crystalline SrTiO_3_/Si pseudo-substrates were fabricated by molecular beam epitaxy (MBE) at ∼400 °C under P(O_2_) ∼5 10^−8^ Torr^31^. SrRuO_3_ bottom electrode was deposited by rf magnetron sputtering at ∼620 °C under P(O_2_) ∼3.7 mTorr in a Ar/O_2_ (10/1) mixture. PZT was deposited by spin-coating from a sol-gel process and crystallized by annealing under oxygen flux at 650 °C^13,30^. Pt top electrodes were deposited by rf magnetron sputtering at room temperature.

### Structural characterization

A high-brilliance X-ray diffractometer with high-resolution (Rigaku SmartLab) equipped with a copper rotating anode and a Ge(220) monochromator with λ_CuKα1_ = 1.54056 Å was used to investigate the structure and the crystalline orientation of the layers. Temperature-dependent diffraction was performed in air using an Anton Paar heater with a graphite dome.

### Ferroelectric characterization

The ferroelectric properties were analyzed along both OOP (in top-bottom configuration) and IP directions by measuring the electric polarization versus electric field (P-E) at room temperature. PUND (Positive Up Negative Down) excitation pulse trains have been used, allowing to discriminate the polarization current from dielectric displacement and leakage currents.

### Pyroelectric characterization

The pyroelectric properties were determined by measuring the remnant polarization (P_r_), from the ferroelectric hysteresis loops (P-E) using PUND method, at different temperatures ranging from 80 K to 300 K, along both the OOP and IP directions.
