# Publisher Correction: Large anisotropy of ferroelectric and pyroelectric properties in heteroepitaxial oxide layers

**DOI:** 10.1038/s41598-018-28053-1

**Published:** 2018-06-22

**Authors:** R. Moalla, S. Cueff, J. Penuelas, B. Vilquin, G. Saint-Girons, N. Baboux, R. Bachelet

**Affiliations:** 1 Institut des Nanotechnologies de Lyon (INL) - CNRS UMR 5270, Univ. Lyon, Ecole Centrale de Lyon, Bâtiment F7, 36 av. Guy de Collongue, 69134 Ecully Cedex, France; 2 Institut des Nanotechnologies de Lyon (INL) - CNRS UMR 5270, Univ. Lyon, INSA de Lyon, Bâtiment Blaise Pascal, 7 avenue Jean Capelle, 69621 Villeurbanne Cedex, France

Correction to: *Scientific Reports* 10.1038/s41598-018-22349-y, published online 12 March 2018

In the original version of the Article, the following text was duplicated in the ‘Results and Discussion’ section:

“(For the out-of-plane (OOP) measurement in a metal-ferroelectric-metal (MFM) structure, the electric field lines pass across the PZT layer vertically toward the bottom electrode, the properties thus obtained, such as P_r_, E_c_, *p* etc, are those of the layer in the direction perpendicular to the surface. For in-plane (IP) measurement, two successive fingers belong to two different combs inversely polarized. Due to the absence of the lower conductive layer, the field lines pass through the PZT layer horizontally from one finger to the next. Properties thus extracted are those of the layer in the direction parallel to the surface. We consider that one of the two combs is the equivalent of the upper electrode of a plate capacitor and the second is the equivalent of the lower electrode, so the distance between two successive fingers is equivalent to the thickness of the layer between the electrodes. The total area of the capacitor is considered as the number of fingers in a comb multiplied by the surface of a finger)”

This has now been corrected in the HTML and PDF versions of the Article.

